# SurvInt: a simple tool to obtain precise parametric survival extrapolations

**DOI:** 10.1186/s12911-024-02475-6

**Published:** 2024-03-14

**Authors:** Daniel Gallacher

**Affiliations:** Warwick Medical School, University of Warwick, CV4 7HL Coventry, UK

**Keywords:** Survival analysis, Interpolation, Extrapolation, Health technology assessment, External data

## Abstract

**Background:**

Economic evaluation of emerging health technologies is mandated by agencies such as the National Institute for Health and Care Excellence (NICE) to ensure their cost is proportional to their benefit. To avoid bias, NICE stipulate that the benefit of a treatment is assessed across the lifetime of the patient population, which can be many decades. Unfortunately, follow-up from a clinical trial will not usually cover the required period and the observed follow-up will require extrapolation. For survival data this is often done by selecting a preferred model from a set of candidate parametric models. This approach is limited in that the choice of model is restricted to those originally fitted. What if none of the models are consistent with clinical prediction or external data?

**Method/Results:**

This paper introduces SurvInt, a tool that estimates the parameters of common parametric survival models which interpolate key survival time co-ordinates specified by the user, which could come from external trials, real world data or expert clinical opinion. This is achieved by solving simultaneous equations based on the survival functions of the parametric models. The application of SurvInt is shown through two examples where traditional parametric modelling did not produce models that were consistent with external data or clinical opinion. Additional features include model averaging, mixture cure models, background mortality, piecewise modelling, restricted mean survival time estimation and probabilistic sensitivity analysis.

**Conclusions:**

SurvInt allows precise parametric survival models to be estimated and carried forward into economic models. It provides access to extrapolations that are consistent with multiple data sources such as observed data and clinical predictions, opening the door to precise exploration of regions of uncertainty/disagreement. SurvInt could avoid the need for post-hoc adjustments for complications such as treatment switching, which are often applied to obtain a plausible survival model but at the cost of introducing additional uncertainty. Phase III clinical trials are not designed with extrapolation in mind, and so it is sensible to consider alternative approaches to predict future survival that incorporate external information.

**Supplementary Information:**

The online version contains supplementary material available at 10.1186/s12911-024-02475-6.

## Background

Emerging health technologies are mandated to demonstrate their clinical and cost-effectiveness by agencies such as the National Institute for Health and Care Excellence (NICE) to ensure their cost is proportional to their benefit. NICE has established thresholds which it compares treatments against to ensure fairness across the consideration of different health technologies and disease areas, and that the National Health Service (NHS) in England and Wales obtains value for money and is able to sustainably provide optimal healthcare.

To avoid bias when appraising a health technology, NICE stipulate that the benefit of a treatment is captured across the lifetime of the patient population, which can be many decades [1]. Unfortunately, follow-up from a clinical trial will not usually provide data for this lifetime period and the observed follow-up will require extrapolation in order for the treatment benefit to be estimated. For a time-to-event outcome, such as death, this is typically done by fitting a parametric model or other model type to the observed data, and extrapolating the model until virtually all patients are predicted to have had the event of interest [2]. A set of candidate models will be fitted to the data, and a preferred model is selected by an assessment of their goodness-of-fit to the observed data and the plausibility of their extrapolations. Plausibility can be assessed through comparison to external data and expert clinical opinion. Alternative plausible models can be used as a form of sensitivity analysis. Uncertainty around a certain may be explored in a probabilistic sensitivity analysis by sampling randomly around the mean parameter estimates using the 95% confidence intervals around the parameters and their correlation [3]. 

Limitations of this approach include the lack of options if no plausible extrapolations are yielded, forcing the modeller to pursue alternative methods, or to compromise on a sub-optimal model. The use of trial data for extrapolation assumes that the observed data will be representative of the routine use of the health technology. This may not be true, particularly in the case where the data has come from a clinical trial with strict inclusion criteria or other carefully controlled conditions, such as treatment switching. It also assumes that the follow-up data are sufficient to produce a model that accurately predicts the future survival of patients, despite the potential for a clear distinction between patients who respond well to therapy whose events are less likely to be observed, and those who do not respond well. It is plausible that neither of these assumptions hold due to the uncertainty of future real-world efficacy and decreasing maturity of trial data included in technology appraisal submissions. Simulations have shown that extrapolation with parametric models can contain bias and/or high uncertainty [4–6]. 

If a clinical trial is not designed with extrapolation in mind, it raises the question of whether current approaches are suitable for providing reliable estimates of effectiveness which contribute to the assessment of cost-effectiveness of a health technology. In fact, simulation studies have shown it may not be, especially when the data are immature [4, 5] which is more likely when the extrapolation is not of data related to a primary outcome. There are alternative methods that utilise external data [7, 8] however these typically rely on access to patient level data, which may not always be available. There are few alternative options, and it is common to proceed with extrapolations even when the data may be ill-suited for such a purpose. This paper introduces an alternative approach to predicting the future survival of a population.

## Implementation

This paper presents SurvInt (https://dgallacher.shinyapps.io/survint/), a R Shiny tool which allows the user to specify population survival at key points and obtain parametric extrapolations that are consistent with those specified by the user. Instead of fitting to survival data, which may not represent real-world use nor provide plausible extrapolations, SurvInt provides a means of obtaining a parametric survival model that is consistent with key desired points that could be based on information from clinical trials, real-world evidence, or expert opinion. When treatment benefit is assessed using life-years and quality-adjusted life-years, it is important to maximise the consistency to sources of information that offer estimates of long-term efficacy. Rather than extrapolating data and hoping for a suitable extrapolation, selecting one point that is consistent with the observed data, such as the median survival or earlier, and a second that comes from external data may produce a more reliable extrapolation when as much as 94% of the treatment benefit is estimated from the extrapolated period and not supported by observed data [3]. 

SurvInt rearranges the survival function and solves it as a series of simultaneous equations using the rootSolve package, interpolating the points specified by the user. SurvInt is currently able to estimate parameters for the exponential, Weibull, log-logistic, log-normal and Gompertz survival models, using the parameterisations as described in the flexsurv Rpackage (https://www.jstatsoft.org/article/view/v070i08). These forms allow a range of varying survival curve shapes and should provide the user with at least one model that is consistent with their data. The exponential distribution requires the user to specify a single point to interpolate, whilst the other parametric models have two parameters to estimate and so require the user to specify two points. In addition, SurvInt provides a visual representation of the resulting survival mode, demonstrating the successful interpolation of the specified point(s). It also allows the user to upload event-time and -type data to overlay a Kaplan-Meier plot to the parametric models to assess visually which model best represents the data. SurvInt Lite is a version of the tool, with fewer features previously known as SurvExtrap (https://dgallacher.shinyapps.io/survint_lite), and will remain freely available.

## Results

SurvInt has at least two areas of application, which are each demonstrated through the following examples.

### Example 1: obtaining consistency with an external data source

There is increasing demand for ways to incorporate into technology assessments information from data registries which boast much larger sample sizes and longer follow-up than clinical trials. However, access to patient level survival data may not be available. Conference abstracts are a common example where patient survival may be minimally reported, e.g. only be reported at 5 and 10 year milestones, without reporting any further information on survival rates at other times [9]. Using SurvInt this information can easily be turned into a range of potential survival extrapolations, or it can be combined with a point estimate taken from an alternative data source, e.g. combining a clinical trial and a historical cohort.

**Fig. 1:**
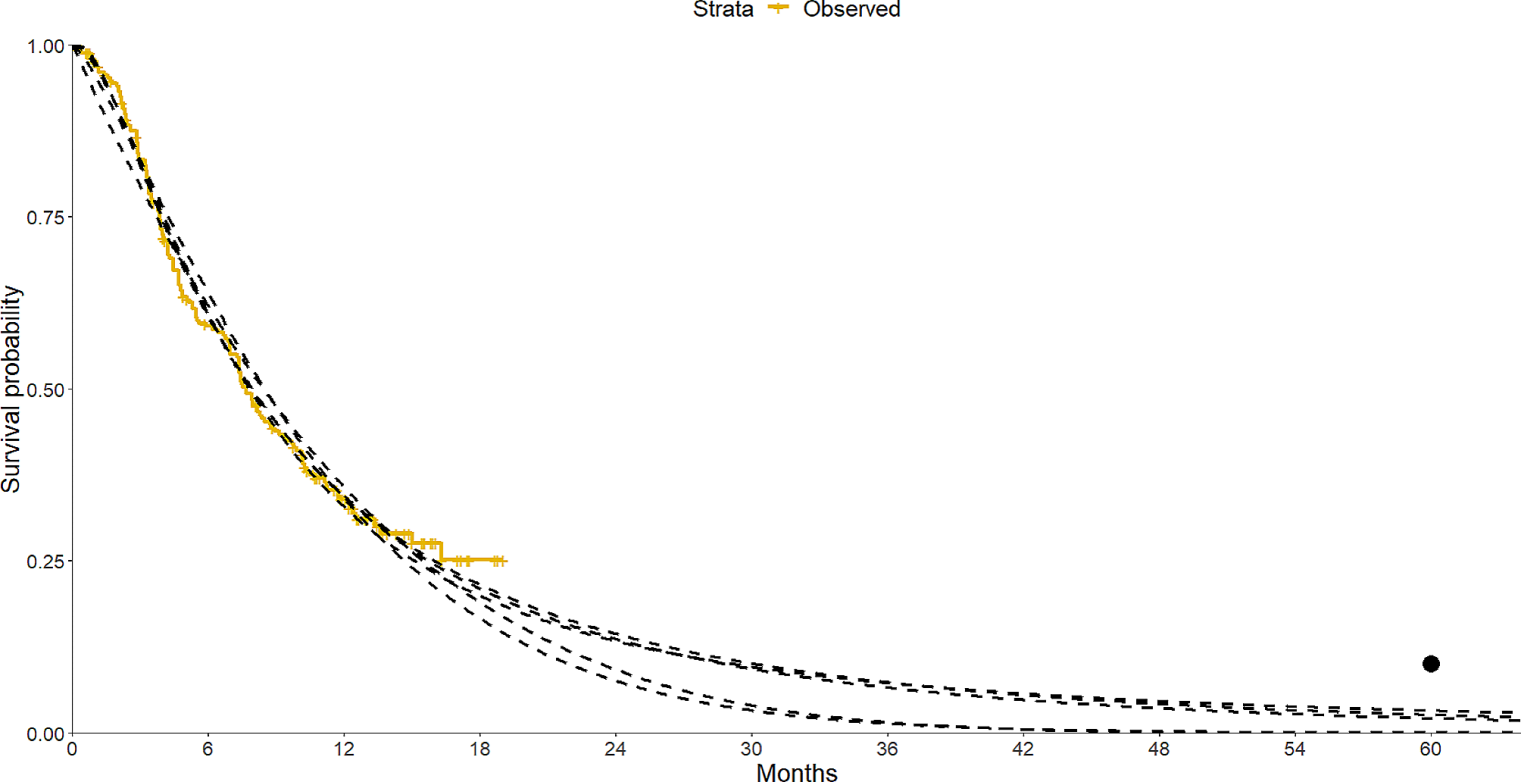
Parametric models fitted to recreated OS data for BSC arm of Keynote-045, with none passing near the circle indicating the 5 year survival rate reported by Cancer Research UK

In the technology appraisal TA519 of pembrolizumab for previously treated advanced or metastatic urothelial cancer, one key discussion point was the survival of the comparator population who received best-supportive care (BSC) [10]. The company’s extrapolations of the BSC data from their KEYNOTE 045 trial produced estimates that disagreed with the 5 year survival rates reported by Cancer Research UK (CRUK). Figure 1 demonstrates a visual representation of the problem, showing the inconsistency of the extrapolations and the CRUK data. This problem persisted even after the company applied an adjustment for the treatment switching that had occurred in the control arm. Whilst this could be explained by differences in baseline characteristics, there was still a desire to use a model that was consistent with the CRUK report, however it was not possible to get a reasonable extrapolation.

**Fig. 2:**
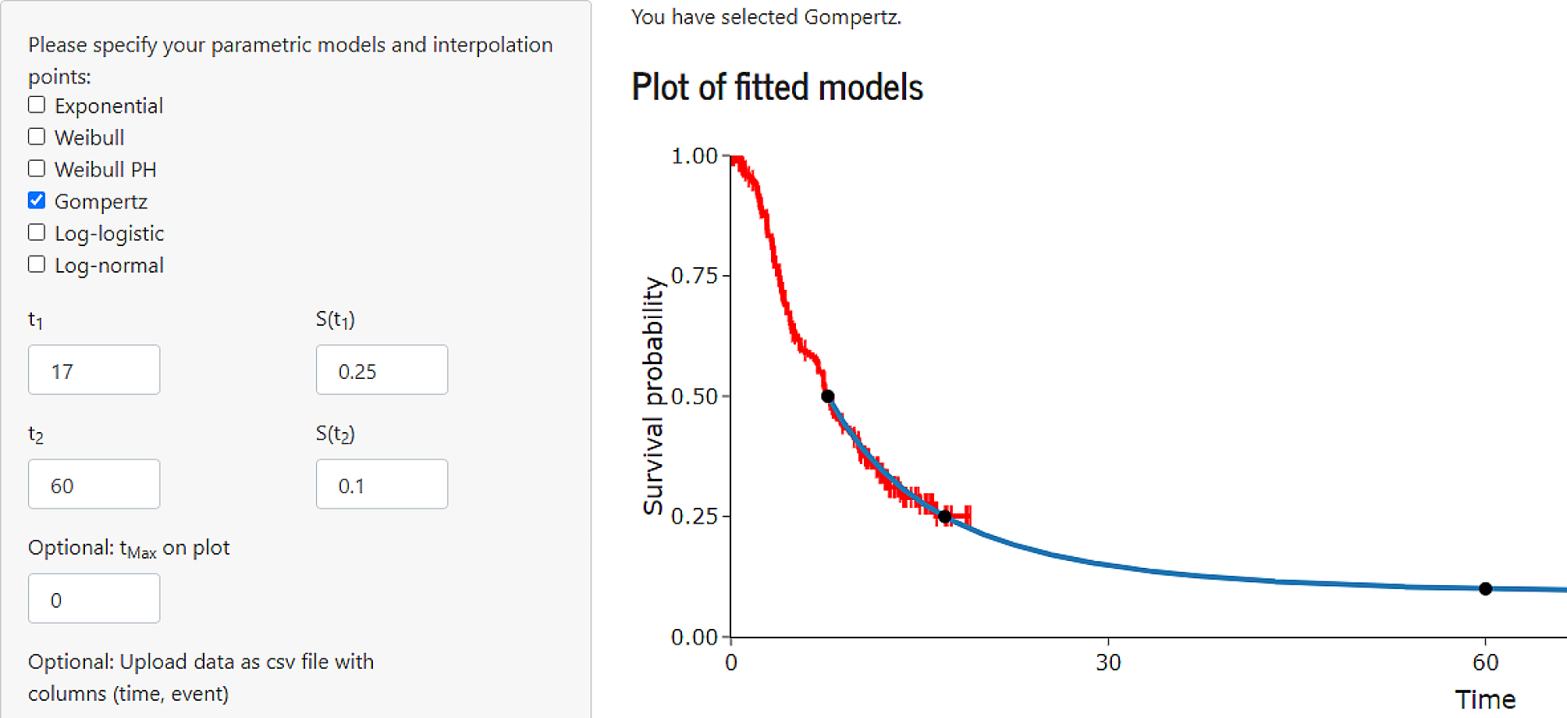
A Gompertz model obtained from SurvInt interpolating the Cancer Research UK 5 year survival rate and the median survival from the data

Using SurvInt to implement a piecewise approach and beginning the extrapolation from the median survival time (7.7 months) allows the user to interpolate a later point of follow-up (17,0.25) and the 5-year CRUK estimate (10%) provides a simple way of obtaining a model that is consistent with the data and with the external source. On this occasion a Gompertz model provided the best visual fit to the data (Fig. 2). The Gompertz model obtained using SurvInt appears an equivalent fit to the models fitted to the data. Any difference in the life-years estimated for the observed period would be negligible, and the reliability of the life-years estimated for the extrapolated period has improved considerably.

### Example 2: exploring uncertainty

Consider the case where the uncertainty associated with the long-term efficacy of a therapy is high, with a wide disparity of estimates made by clinical experts about the survival of patients beyond the observed period. New and emerging cell gene therapies are relevant example of this. Typically, the uncertainty could be explored by exploring the uncertainty around the parameters of a particular model, or by varying the choice of survival model. However, in such a case, these may be unsatisfactory and fail to fully explore the uncertainty expressed by clinical experts. In this example the experts’ predictions are represented visually by full lines, but typically experts would only provide estimates of survival at key follow-up milestones, e.g. 5, 10 and 15 years.

**Fig. 3:**
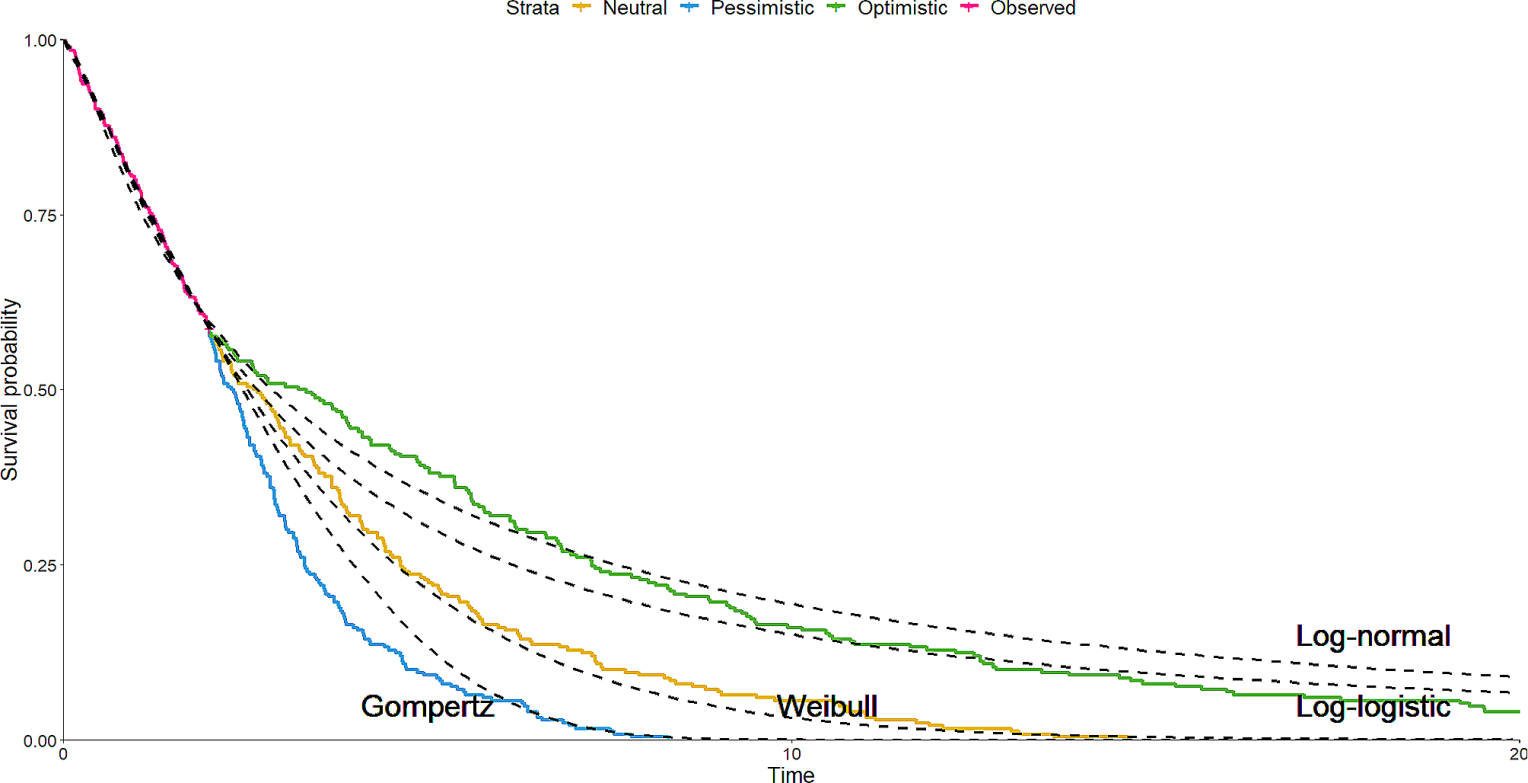
Parametric (dashed) models fitted to observed follow-up compared to predictions made by clinical experts

Using hypothetical data, we show a range of parametric extrapolations (dashed– black) fitted to observed data show by the pink Kaplan Meier curve (Fig. 3). Beyond the observed period, there are three differing opinions on the long-term survival of the patient population. The Weibull model may be selected as it best suits the neutral opinion, but the possibility of the other opinions being right should also be considered. In this case, the Gompertz model fitted to the observed data could be a considered satisfactory to explore a pessimistic scenario, and the two log-models acceptable for an optimistic scenario. The problem with both of these assumptions is that neither are consistent with the opinions provided by the clinical experts. The curves for both scenarios overestimate survival relative to the opinions, the worst violation being the long-term prediction of the log models exceeding the expert’s predictions.

**Fig. 4:**
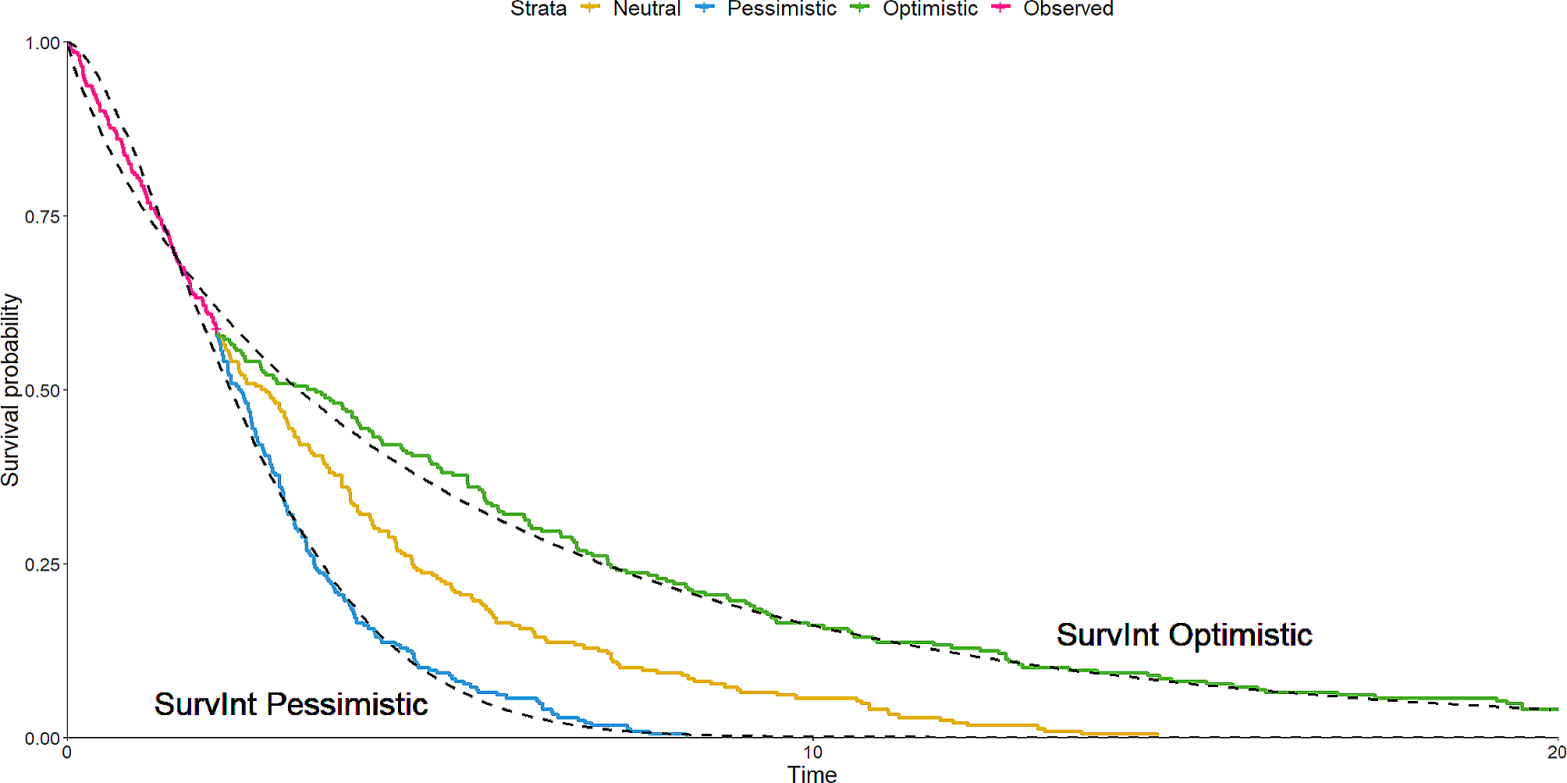
Extrapolations of models with parameter values obtained using SurvInt tool

Using SurvInt, and specifying interpolation of the points S(1.46) = 0.691 and either S(4.73) = 0.101 for the pessimistic scenario or S(12.7) = 0.108 for the optimistic scenario produced estimates of Weibull curve parameters that allowed modelling of the curves seen in Fig. 4. A comparison of the two shows that the models coming from SurvInt are close fits to the predictions made, and are also consistent to the observed data. No great care was taken when selecting these points, and the user could prioritise better fits to earlier or later points, depending on their preference and convergence of the solving algorithm running in SurvInt. In cases where the selected points occur earlier in the follow-up, their specification should be justified, and the robustness of the extrapolations shown through the exploration of parameters obtained from alternative points. A similar approach could be taken to effectively parameterise confidence intervals of the Kaplan-Meier survival function, allowing the exploration of best and worst case scenarios varying assumptions such as the cure fraction.

## Additional features

If none of the candidate models are considered sufficient, SurvInt offers alternative options for the user to explore. Firstly, as shown in the first example, SurvInt can implement a piecewise approach where Kaplan-Meier data are modelled for an initial period, followed by a parametric extrapolation once the data resemble a form that can be captured. The interface allows the user to specify exactly when they would like the parametric model to assume t_0_ occurs, enabling simple avoidance of a region where the Kaplan-Meier survival function is hard to capture with a parametric model.

Secondly, the user can specify to plot the mean average survival estimate of the selected models, ideal when the desired extrapolation sits somewhere between the set of candidate models. The benefits of such an approach for models fitted to data have already been shown [5]. 

The user can also change the scale on x-axis to zoom in or allow consideration of the tail behaviour of the extrapolation, if it is not visible on the default setting. If the extrapolations are too optimistic, then SurvInt allows application of the latest available UK general population mortality rates, to an age and sex matched population specified by the user. This requires the user to also specify the units of time used in their specified interpolation points to ensure that background mortality is correctly applied. If the hazard rate of the parametric model falls below the hazard rate of the general population, then the hazard rate from the general population is applied instead. This adjustment is applied after the parameters are estimated so may cause extrapolations to deviate from the specified points.

SurvInt allows the user to display the hazard rates for the candidate models across the time horizon of the extrapolation, which provide helpful information in selecting a preferred model. Often an increasing/decreasing hazard rate over time can rule out a model on the grounds of clinical plausibility, which may be less apparent when examining on the survival scale.

SurvInt also permits mixture cure models to be implemented. In settings where some patients are considered cured and no longer at risk of an event, the survival curve would demonstrate a plateau that is cannot be well captured by the traditional parametric models. However the mixture model setting allows for the plateau to be accurately modelled. For all models, SurvInt will estimate the restricted mean survival time based on the visible plot region, which can be converted to life-years as used by health economists for valuing treatment benefit.

Finally SurvInt allows the user to explore the range of estimates produced by a probabilistic sensitivity analysis (PSA). If data are uploaded then SurvInt will automatically report the variance and covariance parameters of all the parametric models, fitting them to the uploaded data. These are estimated solely from the uploaded data and are independent from the specified points. If data are uploaded, and only one model is selected, then SurvInt allows the user to run 1,000 PSA iterations and will include the 2.5 and 97.5 percentiles of the extrapolations on the displayed survival plot. The PSA uses the variance-covariance estimates from the data, and applies them with the parameter values estimated by SurvInt that interpolate the required points, in effect using the uncertainty associated with the data as a proxy for the potential uncertainty associated with the extrapolation. There may be cases where this is not sensible, but on the whole, it is not too far removed from the typical approach of assuming the parameter uncertainty applies into the extrapolated period.

**Table 1:** Comparison of SurvInt Features with other recent survival analysis packages

Feature	SurvInt	Standard survival modelling	SurvExtrap [11]	GNOSIS [12]	surviveR [13]	Excess hazard models [14]
Fit model to data	No	Yes	Yes	Yes	Yes	Yes
Combine information from multiple sources (e.g. registry data or background mortality)	Yes	No	Yes (if in correct form)	No	No	Yes
Designed for obtaining extrapolations	Yes	Yes	Yes	No	No	Yes
Available in R Shiny Application	Yes	No	No	Yes	Yes	No
Cure models	Yes	No	Yes	No	No	Yes
Piecewise approach	Yes	No	Yes	No	No	Yes
Suitable with minimal data	Yes	No	No	Yes	Yes	No
Require only basic statistical modelling expertise	Yes	Yes	No	Yes	Yes	No

## Discussion

SurvInt provides the modeller with greater flexibility and freedom to consider any potential extrapolation, releasing them from the typically limited set of parametric models fitted to observed data. This paper has shown two cases where SurvInt can markedly improve the available survival extrapolations which will result in more informative economic analyses. SurvInt cannot tell you which survival model is most appropriate, and this must be assessed through careful consideration of the visual fit to the data and plausibility of the extrapolation. The selection of interpolation points and model shapes should be performed in cooperation with robust evidence sources and expert clinical opinion. Understanding the underlying hazard rate of each model type is also key in selecting the optimal model. As SurvInt is not fitting to data there are no goodness-of-fit statistics to utilise, however the utility of statistics such as AIC and BIC has been shown to be limited [4, 5]. Alternative methods such as dynamic modelling and mixture models may prove to yield improved extrapolations compared to traditional parametric techniques, but still require sufficient follow-up in order for an accurate extrapolation to be obtained [15, 16]. An advantage of SurvInt is that it does not need access to mature follow-up from a single source to obtain a plausible model. SurvInt can be utilised beyond the setting of health technology assessment, where extrapolation of survival from minimal data, such as epidemiological studies. Where data suitable for model fitting are available, approaches such as blended survival models [17] or methods explored by Bullement et al. might be preferred [18]. 

Potential improvements to SurvInt include the addition of alternative parametric models and hazard ratios.

The utility of SurvInt differs from other R Shiny tools focused on survival analysis, such as SurviveR [13] or GNOSIS [12], which allow comparison of survival outcomes for multiple groups, through generation of a Kaplan-Meier plot and log-rank tests or Cox proportional hazards models, and do not consider extrapolation. The recently published survextrap R package does focus on survival extrapolation for economic modelling and allows combination of different sources of data over different timeframes across a variety of modelling approaches, however relies on hazard rate information being available for the alternative source, and is also not currently provided in R Shiny [11]. Sweeting et al. describe methods of incorporating excess hazards into extrapolation models to improve the likelihood of obtaining a plausible extrapolation, also with economic modelling in mind [14]. These and similar approaches can be ideal when you have data suitable for extrapolation, however SurvInt is unique for enabling extrapolations to be obtained from minimal data as it relies on interpolation rather than the fitting of survival models in the traditional sense. A comparison of features is shown in Table 1. A major strength of SurvInt is its simplicity and flexibility, meaning the user does not require advanced knowledge of statistical software to produce a plausible extrapolation from a range of potential scenarios of data availability and survival behaviour. SurvInt is designed with economic modelling in mind, with clear and transparent calculation and reporting of parameter values, alongside the inputs necessary to align with a PSA.

It is important to account for uncertainty associated with survival extrapolations. Statistical uncertainty, as represented in a PSA, relates only to periods of time for which there is observed data, and does not reflect structural uncertainty. The PSA functionality of SurvInt is consistent with existing PSAs in that regard, however allows more precise modelling of specific scenarios of interest. When extrapolations are combined with Kaplan-Meier estimates, there is no established way of factoring in the uncertainty associated with the period relating to the Kaplan-Meier estimation. One potential approach is to bootstrap sample the survival data and refit the Kaplan-Meier model, and combining each iteration with a probabilistic sample for the parameters for the extrapolated period.

Technology appraisal submissions are increasingly reliant on adjustments to populations to account for baseline differences or treatment switching. However, it is rare for the statistical modelling behind these approaches to be reported in sufficient detail for appraisers and decision-makers to be confident in their implementation. Reluctance to share patient data means that the analyses behind these often ad-hoc adjustments are typically more opaque than primary trial analyses. SurvInt could serve as a valuable tool to health-economists when such adjustments are not performed and reported transparently, allowing alternative scenarios to be modelled and their cost-effectiveness impact to be assessed.

## Conclusions

It is vital to be able to estimate the benefit and value of treatments accurately, to ensure current and future healthcare is delivered sustainably. SurvInt offers a simple alternative to parametric extrapolation of data, allowing the exploration of uncertainty and providing a solution in cases where no plausible models are otherwise available. This is helpful when survival information comes from multiple non-combinable sources or is otherwise minimally available. SurvInt allows more precise modelling of treatment benefits and improves the reliability of cost-effectiveness assessments.

## Availability and requirements

Project Name: SurvInt (formerly SurvExtrap).

Project Homepages: https://dgallacher.shinyapps.io/survint/.

Operating System: N/A (R Shiny web application).

Programming Language: R.

Other requirements: None.

License: TBC.

Any restrictions to use by non-academics: licence needed for SurvInt.

### Electronic supplementary material

Below is the link to the electronic supplementary material.

Supplementary Material: A guide to use SurvInt and example dataset.

Supplementary Material 2

Supplementary Material 3

## Data Availability

Not applicable.

## Abbreviations

BSC: Best Supportive Care
CRUK: Cancer Research UK
NHS: National Health Service
NICE: National Institute for Health and Care Excellence
PSA: Probabilistic sensitivity analysis
