# Supplementary material for: SurvInt: a simple tool to obtain precise parametric survival extrapolations
Source: BMC Med Inform Decis Mak. 2024 Mar 14;24:76. doi: 10.1186/s12911-024-02475-6 (PMC10938652; doi:10.1186/s12911-024-02475-6)
Supplement: Supplementary file 1 — Supplementary Material: A guide to use SurvInt and example dataset. [file 12911_2024_2475_MOESM1_ESM.pdf]

SurvInt User Guide

When successfully loading SurvInt, your screen should look like this, without the A-K labelling.

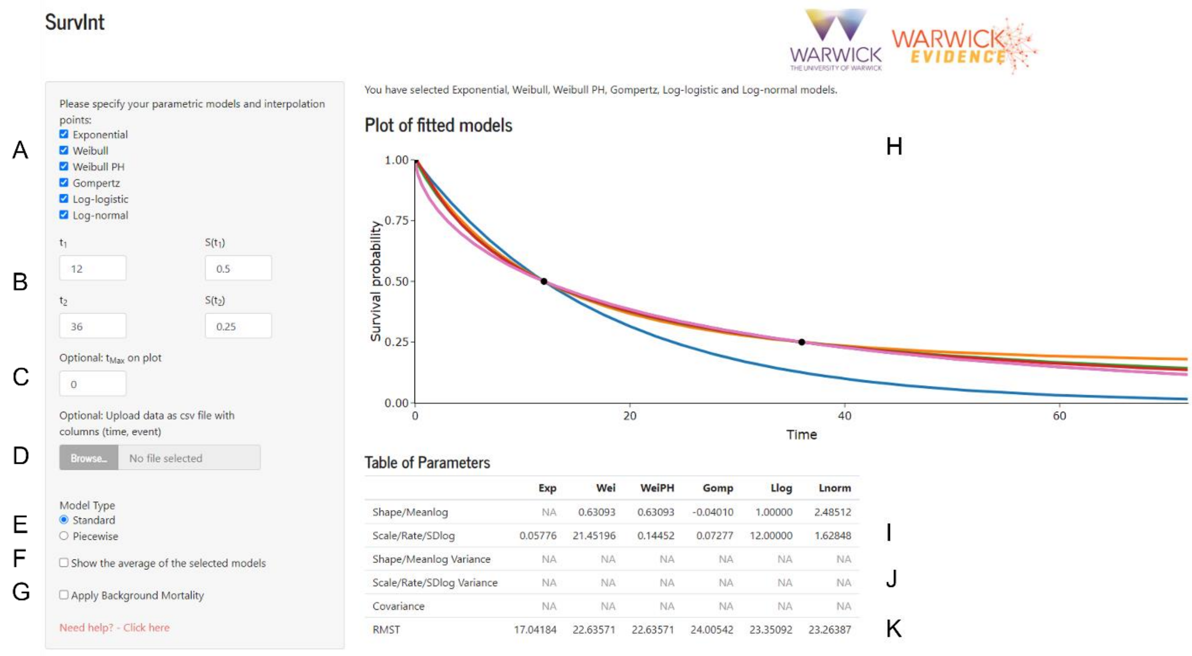

Figure 1: Example of SurvInt

A – Here is where you specify which parametric models you would like to show on the plot. The two Weibull models will have identical forms on the plot, but have different parameterisations. The exponential model will only interpolate the first specified point as it only has one parameter.

B – This is where you specify your co-ordinates $(t_1, S(t_1))$ and $(t_2, S(t_2))$ to be interpolated.

C – Here you can manually specify a maximum value for the time axis on the plot, which is helpful to assess goodness of fit to data, and do investigate behaviour into the future. This also affects the calculation of restricted mean survival time (RMST). By default this value is based on t_2.

D – here you have the option to upload patient level survival data which will display visually on the plot. An example of the format to upload is provided in the supplementary info. This can be helpful in selecting a preferred model. Uploading data is required for the estimation of variance and covariance parameters, and displaying the output from a probabilistic sensitivity analysis (PSA).

E – Here you can switch between standard parametric models, and piecewise models. Standard models will always begin from $t_0 = 0$ and $S(t_0) = 1$. Piecewise models allow the user to specify beginning the parametric model from a later point in time, avoiding a period where the parametric models may struggle to capture the desired survival function. For the missing period, you could model the Kaplan-Meier estimates directly, or use an alternative extrapolation, parametric or otherwise. The parameters are estimated assuming the new user-specified values are t_0 and $S(t_0)$ and so the output will need to be rescaled when implemented in an economic model.

F – Ticking this box adds the plot of the mean average of the selected survival functions. If both Weibull models are specified, then they will be both be included in this average model.

G – Ticking this box will apply background mortality to the extrapolations. This is done by comparing the hazard rate of the extrapolation to the hazard rate of the age and sex matched general population. The hazard rate of the general population is applied if it exceeds the hazard rate from the parametric model. This is applied after the estimation of the parameters and it may cause the models to no longer interpolate the desired points. Background mortality is not accounted for in the PSA.

H – This is the visual output of the extrapolations. The interpolation points are shown alongside all selected extrapolations and the Kaplan-Meier function of the data if uploaded by the user. Hover with your mouse to find out which each extrapolation corresponds to.

I – These are the parameters that correspond to the models interpolate the specified points. They can be used in the parametric forms as implemented and described in the flexsurv R package.

J – These are the variance and covariance for each parameter, which can be used in a PSA. They require the user to upload data, and are calculated from fitting each parametric model to this data. They do not depend on the points specified by the user.

K – This is the restricted mean survival time. If the units of time years, then this is equivalent to life years. They are estimated from the parameters displayed in (I), are calculated based on the area show on the plot (i.e. up to t_{Max}), and do not account for background mortality. This estimate is not reliable when using a piecewise approach.

PSA – By default, the PSA option will not appear. To make it appear, the user must specify just one parametric model and also upload data (see below for an example around the exponential model). If these conditions are satisfied, then the “Run PSA for selected model” option appears. When this is ticked, SurvInt will draw 1000 samples around the mean parameter values that interpolate the points, but use the variance and covariance estimates that come from the relevant parametric model fitted to the data uploaded by the user. One thousand extrapolations will be estimated, and the 2.5 and 97.5 quantiles will be added to the plot, to visualise the uncertainty that would be captured in a PSA if run by the user separately within their economic model. The PSA can take a few minutes, especially if t_{Max} is high. You could consider changing your units of time to speed this up. The PSA does not account for background mortality.

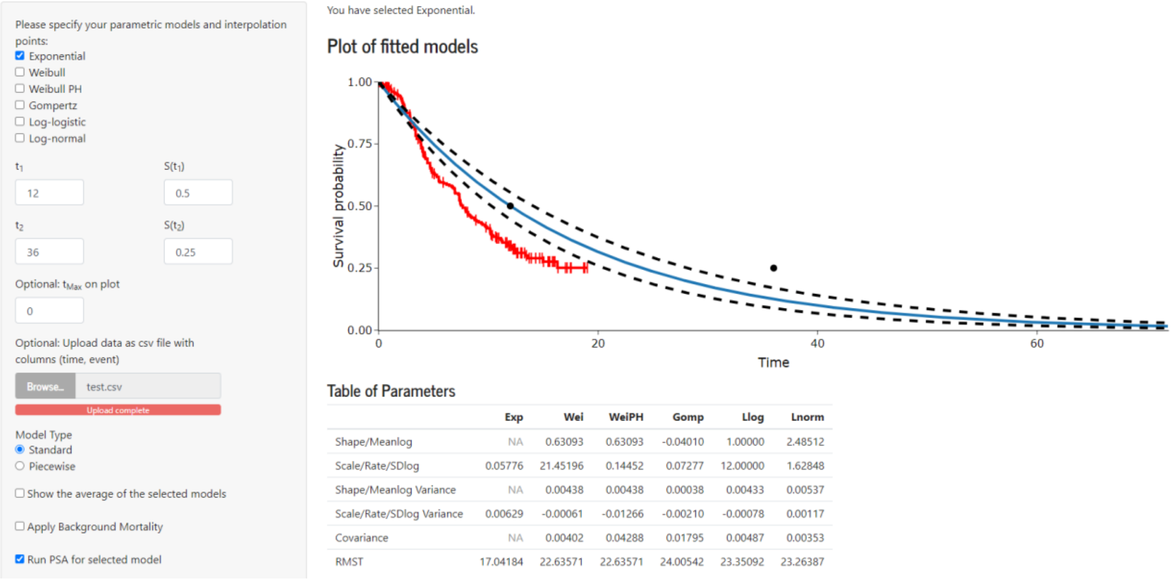

	Exp	Wei	WeiPH	Gomp	Llog	Lnorm
Shape/Meanlog	NA	0.63093	0.63093	-0.04010	1.00000	2.48512
Scale/Rate/SDlog	0.05776	21.45196	0.14452	0.07277	12.00000	1.62848
Shape/Meanlog Variance	NA	0.00438	0.00438	0.00038	0.00433	0.00537
Scale/Rate/SDlog Variance	0.00629	-0.00061	-0.01266	-0.00210	-0.00078	0.00117
Covariance	NA	0.00402	0.04288	0.01795	0.00487	0.00353
RMST	17.04184	22.63571	22.63571	24.00542	23.35092	23.26387

Figure 2: An example PSA for the exponential model. Note it is not meant to reflect the red Kaplan Meier curve
