# Supplementary material for: SurvInt: a simple tool to obtain precise parametric survival extrapolations
Source: BMC Med Inform Decis Mak. 2024 Mar 14;24:76. doi: 10.1186/s12911-024-02475-6 (PMC10938652; doi:10.1186/s12911-024-02475-6)
Supplement: Supplementary file 3 — Supplementary Material 3 [file 12911_2024_2475_MOESM3_ESM.docx]

**Supplementary file legends**

SurvInt User Guide.pdf – User Guide for SurvInt tool

example data.csv – Example data to be uploaded to SurvInt tool
